# Mechanisms of regulated cell death during plant infection by the rice blast fungus *Magnaporthe oryzae*

**DOI:** 10.1038/s41418-024-01442-y

**Published:** 2025-01-10

**Authors:** Matthew R. Wengler, Nicholas J. Talbot

**Affiliations:** The Sainsbury Laboratory, University of East Anglia, Norwich, UK

**Keywords:** Microbiology, Macroautophagy

## Abstract

Fungi are the most important group of plant pathogens, responsible for many of the world’s most devastating crop diseases. One of the reasons they are such successful pathogens is because several fungi have evolved the capacity to breach the tough outer cuticle of plants using specialized infection structures called appressoria. This is exemplified by the filamentous ascomycete fungus *Magnaporthe oryzae*, causal agent of rice blast, one of the most serious diseases affecting rice cultivation globally. *M. oryzae* develops a pressurized dome-shaped appressorium that uses mechanical force to rupture the rice leaf cuticle. Appressoria form in response to the hydrophobic leaf surface, which requires the Pmk1 MAP kinase signalling pathway, coupled to a series of cell-cycle checkpoints that are necessary for regulated cell death of the fungal conidium and development of a functionally competent appressorium. Conidial cell death requires autophagy, which occurs within each cell of the spore, and is regulated by components of the cargo-independent autophagy pathway. This results in trafficking of the contents of all three cells to the incipient appressorium, which develops enormous turgor of up to 8.0 MPa, due to glycerol accumulation, and differentiates a thickened, melanin-lined cell wall. The appressorium then re-polarizes, re-orienting the actin and microtubule cytoskeleton to enable development of a penetration peg in a perpendicular orientation, that ruptures the leaf surface using mechanical force. Re-polarization requires septin GTPases which form a ring structure at the base of the appressorium, which delineates the point of plant infection, and acts as a scaffold for actin re-localization, enhances cortical rigidity, and forms a lateral diffusion barrier to focus polarity determinants that regulate penetration peg formation. Here we review the mechanism of regulated cell death in *M. oryzae*, which requires autophagy but may also involve ferroptosis. We critically evaluate the role of regulated cell death in appressorium morphogenesis and examine how it is initiated and regulated, both temporally and spatially, during plant infection. We then use this synopsis to present a testable model for control of regulated cell death during appressorium-dependent plant infection by the blast fungus.

## FACTS

- Regulated cell death is critical for appressorium morphogenesis in the rice blast fungus
- Regulated conidial cell death requires autophagy and is necessary for appressorium re-polarization and pathogenesis
- Autophagy is associated both with conidial cell death and the generation of a functionally competent appressorium
- Conidial cell death is reported to require ferroptosis and involve lipid peroxidation

## OPEN QUESTIONS

- How are the individual fates of conidial cells regulated both temporally and spatially?
- What is the precise nature of the pro-death signal in conidial cells of the blast fungus?
- What is the role of TOR signalling in the regulation of autophagy and conidial cell death?
- How is autophagy required both for cell death and for invasive growth by the blast fungus?

## Introduction

Filamentous fungi and oomycetes are unique among microbial pathogens in their ability to rupture the external cuticle of plants using specialized infection structures called appressoria. This enables them to invade plant tissue efficiently, which partly explains their success as plant pathogens. Fungi and oomycetes are, for example, responsible for many of the world’s most devastating crop diseases and their control using fungicides or resistant crop varieties constitutes one of the biggest challenges to global agriculture. The cost of controlling potato late blight alone, for instance, and associated yield losses is up to $10 billion per annum [1]. Furthermore, despite these interventions plant pathogenic fungi still cause 10–20% of crop losses each year [2]. Understanding the developmental biology of plant pathogenic fungi is therefore important if new and durable means of disease control are to be discovered.

Recently, it has become clear that regulated cell death plays a pivotal role in infection-related development by fungi. To form an appressorium [3], fungal spores undergo a type of regulated cell death that is necessary for plant infection to proceed [4], while in necrotrophic fungi a distinct type of programmed cell death is associated with host tissue colonization [5]. In this review, we describe recent progress in our understanding of the mechanisms and control of fungal regulated cell death and its role in plant infection. We focus primarily on investigations in the rice blast fungus *Magnaporthe oryzae* (syn. *Pyricularia oryzae*), a model system for understanding plant infection processes [6–8] where studies to date are the most advanced.

**Fig. 1:**
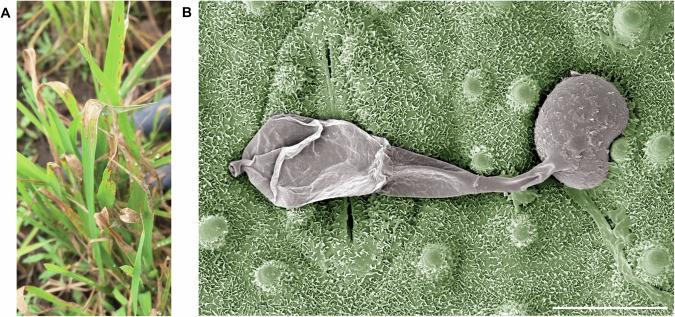
The filamentous ascomycete fungus *Magnaporthe oryzae* is the causal agent of rice blast disease. **A** Rice blast is characterized by large, necrotic lesions appearing on all above-ground parts of the plant. **B** False colouring of a scanning electron micrograph showing a collapsed conidium that has undergone regulated cell death (left) and the dome-shaped pressurized appressorium (right) on the rice leaf surface. A rice leaf stoma is shown beneath the conidium and the hydrophobic waxy cuticle is evident. Scale bar = 10 µm.

Rice blast disease is the most serious threat to the global rice production, destroying sufficient rice each year to feed 60 million people [9, 10], but the fungus can also infect over 50 different grass species, including wheat, finger millets, and barley [11, 12]. *M. oryzae* differentiates a dome-shaped appressorium to gain entry to plant tissue (Fig. 1) [13]. The appressorium develops enormous turgor pressure by accumulating osmolytes, such as glycerol, and drawing in water from dew drops on the leaf surface [14–17]. Repolarization of the appressorium directs turgor to its base, resulting in emergence of a rigid penetration peg that mechanically ruptures the host cell wall [18]. Once the epidermal cell is breached, the penetration hypha differentiates into bulbous invasive hyphae that are encased in the invaginated host plasma membrane [19]. As the fungus invades plant tissue it secretes a repertoire of virulence proteins, called effectors, which manipulate host cell physiology and suppress plant immunity [20]. The fungus colonizes neighbouring cells via plasmodesmata and initiates a necrotrophic phase of infection, inducing cell death and macerating host tissues [8]. Conidia are then produced from disease lesions and dispersed to neighbouring host plants to initiate new infections.

Appressorium development is regulated by signal transduction pathways that monitor the prevailing physical and nutritional environment, and metabolic status of the pathogen. The mitogen-activated protein kinase (MAPK) Pmk1 signalling pathway is activated in response to surface hydrophobicity [21] and acts downstream of, or in tandem with, the cyclic AMP-dependent protein kinase A (PKA) signalling pathway to initiate appressorium development [22–25]. Upon germ tube extension, the nucleus in the apical conidial cell undergoes a single round of mitosis and a resulting daughter nucleus is trafficked to the incipient appressorium, while the other nucleus re-enters the conidium [4]. An S-phase checkpoint is required to initiate appressorium development, while entry into G1 is necessary for appressorium maturation, and mitotic exit is required for functional competence of the appressorium [26]. Additionally, it has been reported that cell cycle arrest at G2 is required for appressorium maturation, metabolically regulated by the Target of Rapamycin (TOR) signalling pathway [27]. The three-celled conidium undergoes autophagy and sequential cell death to supply the appressorium with cytosolic materials required for appressorium turgor generation and invasive growth [4, 28]. Autophagy and regulated cell death of the conidium are therefore prerequisites for rice blast disease but, despite an expanding understanding of the regulatory networks that initiate appressorium development, several questions remain unanswered.

## Mechanisms of autophagy in *Magnaporthe oryzae*

Autophagy is a conserved catabolic process that maintains intracellular homoeostasis during development and in response to environmental stress. Cytoplasmic contents destined for degradation are sequestered by a double membraned cup-shaped structure called a phagophore, which expands to encapsulate its cargo that may include cytoplasm and/or organelles [29]. Following sequestration, the double-membrane vesicle, now termed an autophagosome, is transported to the vacuole with which it fuses [30]. Upon vacuolar fusion, the encapsulated contents are released from autophagic bodies, degraded by vacuolar hydrolases, and subsequently exported for recycling. Starvation-induced autophagy (sometimes referred to as cargo-independent or non-selective autophagy) is induced in response to starvation conditions to recycle bulk cytosolic materials [31]. By contrast, selective autophagy (also referred to as cargo-dependent autophagy) can be induced in the absence of starvation to selectively degrade damaged proteins or organelles, and thereby maintain intracellular homoeostasis.

To control autophagy, more than 40 autophagy-related genes (*ATG*) were initially identified in yeast and have orthologs in many eukaryotes [32]. A set of core Atg proteins is necessary for both starvation-induced and cargo-dependent autophagy and can be divided into sub-groups based on their functional role in autophagosome formation and maturation [29]. The Atg1/ULK complex (Atg1, Atg11, Atg13, Atg17, Atg29 and Atg31) provides an initial scaffold essential for autophagosome initiation at the phagophore assembly site. A membrane delivery cycling system (Atg2, Atg9, and Atg18) plays a role in recruiting membranes to assist phagophore expansion. The phosphatidylinositol 3-kinase complex (Vps34, Vps15, Vps30/Atg6, and Atg14) then assembles during vesicle nucleation to recruit phosphatidylinositol-3-phosphate-binding proteins to the phagophore site, and two ubiquitin-like conjugation systems (Atg5, Atg7, Atg10, Atg12, and Atg16; Atg3 Atg4, Atg7 and Atg8) are subsequently required for vesicle expansion.

Comparative genome analysis between *M. oryzae* and *S. cerevisiae* has identified 23 autophagy-related genes in the blast fungus, which can be functionally annotated based on their roles in either cargo-independent or cargo-dependent autophagy [28]. Deletion of any of the *M*. *oryzae ATG* genes involved in cargo-independent autophagy including those involved in autophagy initiation (*ATG1* and *ATG17*), nucleation (*ATG6*), phagophore and autophagosome expansion (*ATG3, ATG4, ATG5, ATG7, ATG8, ATG10, ATG12* and *ATG16*), and recycling (*ATG2, ATG9, ATG15*) result in loss of pathogenicity. *ATG14*, a component of the phosphatidylinositol 3-kinase complex, more recently identified in *M. oryzae* based on amino acid homology with its yeast counterpart is also indispensable for infection [33]. Notably, core components of the yeast autophagic machinery *ATG13* and *ATG18* are dispensable for infection [28, 34]. Several functional studies have since highlighted the conservation of autophagic machinery between *M. oryzae* and yeast. For example, *M. oryzae ATG1, ATG4*, and *ATG5* can complement defects of respective yeast mutants [35–37].

By contrast, deletion of genes required for cargo-dependent autophagy (*ATG11, ATG24, ATG26, ATG27, ATG28*, and *ATG29*) does not impact pathogenicity [28, 38], although two reports [39, 40] have suggested that cargo-dependent autophagic degradation of dysfunctional mitochondria, regulated by Atg24, is required for appressorium development and invasive growth. Nevertheless, the authors acknowledge that penetration rates of the appressorium are restored in a Δ*atg24* mutant by 48 hpi, although the rate of secondary hyphae development is reduced. Therefore, while it is likely that cargo-dependent autophagic degeneration of mitochondria may aid appressorium maturation and tissue colonization, it is not specifically required for pathogenicity.

## The physiological role of autophagy and cell death in *Magnaporthe oryzae*

**Fig. 2:**
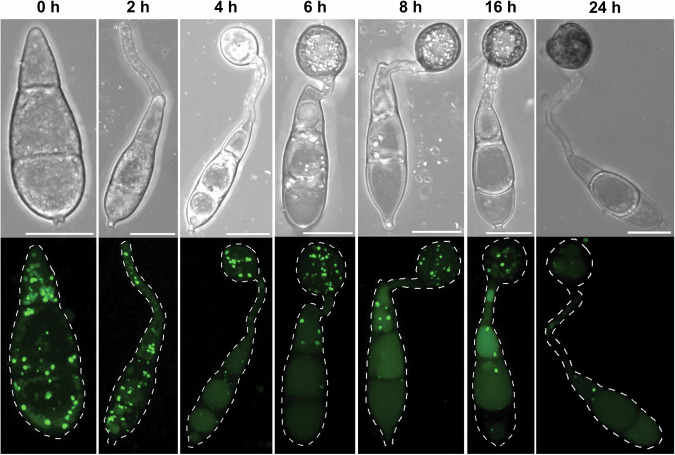
Live cell imaging to show localization dynamics of autophagosomes during appressorium development by *Magnaporthe oryzae.* Maximum projection Z-stack micrographs of autophagosome localization visualized by expression of GFP-Atg8 in the wild type *M. oryzae* strain Guy11. Conidia were inoculated on hydrophobic glass coverslips, allowed to develop appressoria, and observed by confocal microscopy at the indicated times during appressorium development. Scale bar = 10 µm.

Infection-associated conidial cell death in *M. oryzae* has been proposed to be necessary for generation of appressorium turgor and to fuel fungal growth before successful acquisition of nutrients from the plant host [4, 28]. Appressoria generate mechanical force to rupture the rice leaf cuticle and turgor is generated by accumulation of intracellular glycerol within the appressorium to create up to 8.0 MPa of pressure. The nutrient-free environment of the leaf surface therefore requires the fungus to recycle and mobilize storage compounds from the conidium to the incipient appressorium. The number of autophagosomes observed within the conidium peaks at the point of spore adhesion and gradually decreases during appressorium development (Fig. 2). Fungal storage products in *M. oryzae* spores include glycogen, lipid droplets, mannitol, and trehalose [17], but the precise precursors necessary for glycerol biosynthesis remain unclear. Redundancy in triacylglycerol lipase-encoding genes has challenged efforts to assess their relative contribution to glycerol production, although lipid degradation and fatty acid metabolism are clearly critical to appressorium function [41]. The fungal glyoxylate cycle is also pivotal to successful rice blast infection [42], while degradation of storage carbohydrates does not seem to play as major a role in turgor generation [17]. Cargo-independent autophagic breakdown and mobilization of these storage products are necessary for turgor generation and mutants lacking cargo-independent autophagic machinery retain lipid or glycogen reserves within the conidium resulting in reduced turgor generation and are attenuated in pathogenicity [33, 35, 36]. This process also requires cAMP-dependent protein kinase A activity [43] and *ΔcpkA* mutants develop small, non-functional appressoria.

Following germination, the nucleus of the apical conidium cell migrates into the germ tube and undergoes mitosis. One of the daughter nuclei is trafficked to the developing appressorium, while the other returns to the apical conidium cell [44]. Mitosis is followed by an asymmetric cytokinesis resulting in differentiation of the appressorium. As the appressorium develops, each cell of the conidium undergoes sequential rounds of cell death. Nuclear degeneration is observed beginning with the distal cell, until only the appressorium nucleus remains by 24 h post germination. Unlike yeast, *M. oryzae* nuclear degeneration of conidium cells does not depend on microautophagy of the nucleus [45] but instead requires cargo-independent autophagy. Mutants lacking *ATG1* or *ATG4*, for instance, do not undergo nuclear degeneration, fail to penetrate their host, and are attenuated in their ability to undergo vacuolar degeneration [46].

## Regulation of autophagy in *M. oryzae* during infection-related development

How the initiation of autophagy and cell death are regulated in *M. oryzae* is not well understood, but several lines of evidence suggest involvement of the TOR signalling pathway. In yeast, the TOR kinase forms two functionally and structurally distinct complexes named TORC1 and TORC2 which integrate metabolic status and environmental stress with anabolic and catabolic cellular processes to maintain growth and homoeostasis [47], and it has been proposed that both complexes are likely to be conserved across eukaryotes. Several upstream regulators of TORC1 have been identified, including a subset of amino acids and nucleotides, which likely co-regulate homoeostasis under variable conditions. Following exposure to starvation stress, for example, TORC1 is inactivated and the phosphorylation-dependent repression of Atg13 is alleviated, enabling formation of the Atg1-Atg13 complex to initiate autophagy [48]. In contrast, the role of TOR signalling in the pathogenicity of filamentous fungal and oomycete plant pathogens has only recently been investigated. TOR signalling appears to be essential for vegetative growth and treating spores with rapamycin suppresses pathogenicity across several plant pathogens (see Table 1) [49–57]. Transcriptomic analyses have meanwhile linked TOR signalling to autophagy, ribosomal biogenesis, and the regulation of cell wall degrading enzymes [50, 54].

**Table 1:** Inhibition of the TOR kinase suppresses pathogenicity in plant pathogenic fungi.

Pathogen	Host	Treatment	Reported phenotype	Reference
*Botrytis cinerea*	Apple, pear, grape	Rapamycin	Reduced pathogenicity	[55]
	Arabidopsis, potato, tomato	Rapamycin	Reduced conidiation, reduced pathogenicity	[54]
*Fusarium oxysporum*	Potato	RNA interference	Reduced pathogenicity	[56]
*Magnaporthe oryzae*	Rice	Rapamycin	Reduced pathogenicity	[61]
*Phytophthora infestans*	Potato	AZD8055	Reduced pathogenicity	[49]
*Sclerotinia sclerotiorum*	Soybean, kidney bean, tomato, pepper	RNA interference	Reduced pathogenicity	[57]
*Verticillium dahliae*	Cotton	Rapamycin	Reduced pathogenicity	[50]

In *M. oryzae*, processes targeted by TOR include cAMP/PKA signalling, glucose and glutamine sensing, and the cell wall integrity pathway [27, 58–60]. The modulation of TOR signalling likely plays a pivotal role in the regulation of autophagy during infection-related development. For example, the epigenetic regulator Snt2 is positively regulated by TOR and functions to regulate the expression of autophagy genes through histone H3 acetylation [61]. Snt2 activity promotes the expression of genes required for ribosome biogenesis and translation initiation while suppressing the expression of genes involved in autophagy.

**Fig. 3:**
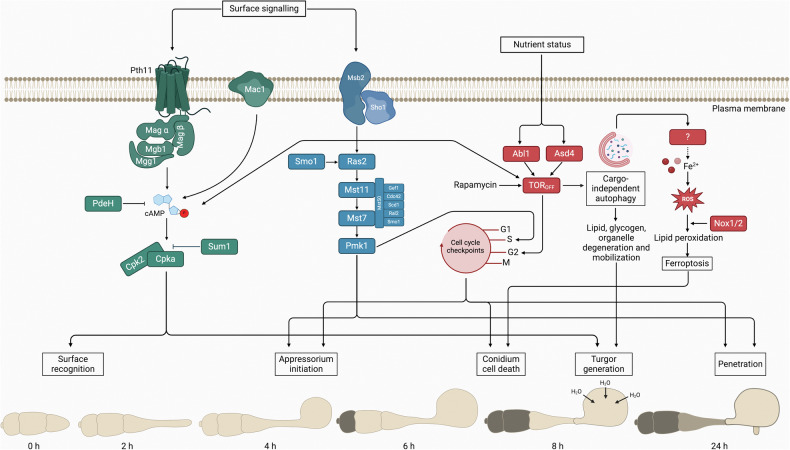
Model for the mechanism by which cAMP/PKA, Pmk1, and TOR signalling pathways regulate regulated cell death and appressorium-mediated plant infection by *M. oryzae.* Appressorium initiation and conidium cell death are regulated by three core signalling pathways; the cAMP/PKA, Pmk1, and TOR signalling pathways. Surface cues, including hydrophobicity and surface hardness, are perceived by upstream components of the cAMP/PKA and Pmk1 signalling pathways, Pth11, Msb2, and Sho1. Activation of cAMP/CPKA is required for surface recognition and turgor generation. Pmk1 MAPK activation is necessary to initiate appressorium development, requiring an S-phase cell-cycle checkpoint. The absence of nutrients on the host surface suppresses TOR activity (TOR_OFF_) via glucose- and glutamine-responsive Abl1 and Asd4, respectively. TOR inactivation arrests the cell cycle in G2 to initiate appressorium maturation and de-represses cargo-dependent autophagy. The autophagic breakdown and mobilization of conidial storage products into the appressorium cell supplies the structure with substrates for turgor generation. The autophagic-dependent release of iron (from an unknown source) then facilitates production of reactive oxygen species (ROS) which leads to generation of lipid peroxides to lethal concentrations, leading to ferroptotic cell death, which is necessary for appressorium function. Figure created with BioRender.com (https://BioRender.com/p83g790*)*.

At the onset of germination, the nutrient-limiting conditions of the leaf surface are likely to suppress TOR activity, and this has been proposed to be a mechanism that leads to arrest of the cell-cycle at G2 as shown in Fig. 3 [27]. In this model, a metabolically regulated cell-cycle checkpoint is proposed to be necessary for initiation of appressorium morphogenesis and autophagy. TOR inactivation is anticipated to be maintained by the Asd4 GATA transcription factor which represses expression of genes involved in nitrogen assimilation to maintain low glutamine levels [58]. Similarly, the glucose-responsive Abl1 protein maintains TOR in its inactive state in the absence of exogenous glucose [27]. Constitutive TOR activation in *Δasd4* or *Δabl1* mutants, or in spores treated with glucose, thereby results in accelerated rounds of mitosis and septation, loss of autophagy, and inhibition of appressorium development [27, 58]. Treating *Δabl1* mutants or glucose-treated spores with the G2 inhibitor benomyl, or rapamycin, prior to G2 has been reported to rescue appressorium initiation and autophagy [27]. Autophagic liberation of endogenous TOR-activating substrates, such as glucose and glutamine, may therefore re-activate TOR to force mitotic progression through M phase and subsequently G1 arrest of the appressorium nucleus to limit continued rounds of mitosis, which is known to further require an S phase checkpoint to enable appressorium re-polarization [62].

Although rapamycin treatment rescues appressorium initiation in spores with inappropriate TOR activation [27], appressoria fail to mature [61]. Early reports demonstrated that spores treated with 200 nM rapamycin induced appressorium initiation under non-inductive conditions and underwent conidium cell death [27]. However, later reports demonstrated that treating with 1 µg/mL (1.094 µM) rapamycin reduced the rate of conidium cell death, inhibited the degradation and mobilization of conidium glycogen and lipid reserves into the appressorium, and reduced pathogenicity [61]. It is possible that nanomolar concentrations of rapamycin may be sufficient to mimic a threshold of TOR inactivation required for appressorium initiation (otherwise mediated through the absence of nutrients on the host surface). Whereas micromolar concentrations of rapamycin maintain TOR inactivation beyond a threshold, resulting in the overstimulation of autophagy, trapping of storage products within the conidium, and suppressing cell death. Why the overstimulation of autophagy inhibits cell death is currently not clear and may provide evidence that a “pro-death” signal mediating conidium cell death involves processes other than autophagy.

During invasive growth TOR is proposed to continue to fluctuate between states of inactivation and autophagy-dependent reaction to promote rounds of mitosis, the maintenance of the biotrophic interfacial complex, and cell-to-cell movement [63]. Upon penetration, a metabolic transition in the fungus occurs switching from dependence on lipid metabolism to glucose metabolism. The transketolase enzyme (*TKL1*) traffics sugar phosphates into glycolysis resulting in production of NADPH and ATP [64]. Through an unknown mechanism, increased ATP levels may lead to reactivation of TOR to promote migration of the appressorium nucleus into invading hyphae by 24 hpi. Immediately following penetration, poor host nutrient acquisition may inactivate TOR, inducing autophagy and halting further growth by 28 hpi [65]. Glutamine and glutamate pools liberated by autophagy are then converted into α-ketoglutarate via glutaminolysis and the (glutamine synthase-glutamine oxoglutarate aminotransferase (GS-GOGAT) pathway. The serine/threonine kinase Rim15 regulates expression of genes involved in these metabolic pathways as well as autophagy induction in a process parallel to, but independent of TOR. Autophagy induction is also dependent on sourcing membranes from endosomes and the plasma membrane by Imp1 which mediates phagophore expansion [66]. Moreover, Imp1 is required for vacuolar V-ATPase assembly and downstream organelle acidification. By 32 hpi, the biotrophic interface is formed and accumulation of α-ketoglutarate stimulates TOR re-activation to promote cell-cycle progression and continued elaboration of hyphae. By 36 hpi, amino acid depletion is proposed once again to drive TOR inactivation and thereby promote autophagy. By 44 to 48 hpi, invasive hyphae migrate into neighbouring plant cells in a Pmk1-dependent process involving development of a specialised transpressorium [8, 67]. Interestingly, both Rim15 and Imp1 are required for maintenance of the extra-invasive hyphal membrane that encases invasive hyphae within plant cells and the biotrophic interfacial complex (BIC), a plant membrane-rich structure implicated in effector deliver to host cells [68]. Both *Δrim15* and *Δimp1* mutants are perturbed in plasma membrane homoeostasis, resulting in aberrant release of the apoplastic effector Bas4, loss of an observable BIC, and attenuation of cell-cell movement by the fungus [66].

A feed-forward network between glucose-sensing and surface-sensing mediated by TOR and cAMP/PKA signalling has also been suggested which stimulates appressorium initiation and activation of autophagy [59]. Upon exposure to hydrophilic surfaces or glucose treatment, spores have been reported to undergo accelerated rounds of mitosis within an extended germ tube and fail to undergo autophagy. It has been proposed that cell-cycle arrest at G2 mediated by TOR inactivation and exposure to cAMP are both required to induce appressorium formation. Consistent with this model, *Δabl1* mutants treated with cAMP or *Δmac1* mutants exposed to rapamycin are not sufficient to initiate appressorium formation. Under unfavourable conditions, activation of TOR and suppression of cAMP/PKA signalling may therefore facilitate germ tube growth and accelerated rounds of mitosis until inductive conditions for appressorium initiation are sensed [59].

In addition to the cAMP/PKA signalling pathway, surface hydrophobicity is monitored by the Pmk1 mitogen-activated protein kinase (MAPK) pathway [25]. Spores incubated on hydrophilic surfaces or disrupted in Pmk1 MAPK function, develop undifferentiated germlings and fail to undergo autophagy. Pmk1 activation is regulated by upstream membrane-localized Msb2 and Sho1 proteins which act via the Mst11 MAPKK kinase and Mst7 MAPK kinase to phosphorylate Pmk1 [69]. Pmk1 activation then results in phosphorylation and activation of a hierarchical transcriptional reprogramming involving transcription factors Hox7 and Mst12 [21, 23] and phosphorylation of regulatory proteins such as Vts1 [21]. A subset of genes involved in autophagy and cell-cycle progression are differentially regulated by Pmk1 during appressorium morphogenesis. Interestingly, Pmk1 likely acts independently of TOR because *Δpmk1* and *Δhox7* mutants treated with rapamycin or benomyl fail to initiate appressorium formation or undergo autophagy.

## Regulated cell death in *Magnaporthe oryzae* may require ferroptosis

Evidence has recently been presented that conidium cell death occurs via ferroptosis (Fig. 3) [70], in which iron-dependent reactive oxygen species production leads to accumulation of intracellular lipid peroxides to lethal concentrations in the conidium [71]. Although characterized extensively in mammals, the final executioner of ferroptosis remains unknown and cell death likely occurs due to destabilization of the plasma membrane, resulting in osmotic swelling and rupture [72]. Under normal physiological conditions, detoxification of lipid peroxides occurs via glutathione peroxidases which utilize reduced glutathione as a cofactor [73]. Iron chelation or lipophilic antioxidants inhibit ferroptosis while genetic or pharmacological inactivation of the lipid peroxide detoxification system leads to its strong induction [71, 74]. It has been reported that *M. oryzae* conidia treated with the iron chelators ciclopirox olamine (CPX) or deferoxamine do not undergo cell death, whereas application of exogenous iron (FeCl_3_) advances cell death [70]. Iron accumulation in the terminal conidium cell is observed by 7 hpi coinciding with the onset of cell death. Moreover, conidia treated with lipophilic antioxidants, liproxstatin-1 (Lip-1) or ferrostatin-1, fail to undergo cell death. Lipid peroxidation has been further quantified in vivo using C11-BODIPY^581/591^ fluorescent probes which shift from red to green upon oxidation. Green fluorescence was observed at the plasma membrane of the terminal conidium cell at 7 hpi but was absent in the other two conidial cells. Fluorescence was meanwhile attenuated in conidia treated with Lip-1, whereas conidia exposed to buthionine sulfoximine, an inhibitor of glutathione biosynthesis, exhibit complete peroxidation. One of the mechanisms for lipid peroxidation initiation includes NADPH oxidase-mediated ROS production. The rice blast fungus encodes three NADPH oxidases with the potential to generate lipid peroxides. Nox1 and Nox2, for example, have previously been shown to regulate septin-mediated cytoskeletal remodelling in appressoria required for infection [75]. Genetic or pharmacological inhibition of these NADPH oxidases reveals reduced conidial cell death and lipid peroxidation at the plasma membrane [70]. Blast infection is significantly delayed in conidia treated with Lip-1 or CPX prior to inoculation. By contrast, exogenous application of iron advanced development of invasive hyphae. As observed in mammalian systems [76], Shen and colleagues [70] report that autophagy likely modulates iron availability to regulate ferroptosis. In wild type spores, for instance, cell death of the distal conidium cell occurs around 8 hpi, whereas cell death of the distal cell in a *Δatg8* mutant is not observed until 24 hpi when a small percentage of cells collapse. The *Δatg8* mutant exhibits reduced iron accumulation and lipid peroxidation, further indicating that ferroptosis is an autophagy-dependent process.

In mammals, there is mounting evidence for a central role for mitochondrial metabolic function and ROS production in regulation of ferroptosis [77, 78]. Inhibition of the TCA cycle, disrupted electron transport chain function, or depolarization of the mitochondrial membrane potential all inhibit ferroptosis. New pre-printed results propose that cargo-dependent autophagic degradation of dysfunctional mitochondria is required for ferroptosis and blast infection [40]. Mitochondrial degeneration during appressorium development is sequential, beginning with the distal conidium cell before progressing to the middle and apical cells (A. B. Eseola and N. J Talbot, unpublished). This pattern coincides with the onset of cell death and Shen and colleagues have investigated the relationship between mitochondria dynamics and ferroptosis [40]. Knocking out *ATG24* resulted in attenuated pathogenicity [39] although this contrasts with previous analysis of its function in autophagy in *M. oryzae* [28]. Disrupting the autophagic degeneration of dysfunctional mitochondria by deleting *ATG24* would be predicted to affect mitochondrial membrane potential and inhibit the accumulation of iron, lipid peroxidation, and the occurrence of cell death, according to the model proposed by Shen and colleagues [40]. Likewise, disrupting mitochondrial membrane potential by inhibiting the electron transport chain Complex III or depolarizing membrane potential by treating with the membrane uncoupler FCCP would also be predicted to decrease the occurrence of cell death.

In addition to iron accumulation and lipid peroxidation, several hallmarks of ferroptosis have been established in *M. oryzae* [79]. Accumulation of autophagosomes in ferroptotic cells, for example, is a morphological hallmark also observed in conidia during appressorium development [28]. Other transcriptional and proteomic biomarkers exist, including genes or gene products involved in production of lipid peroxide precursors, glutathione metabolism, and expression of transcription factors required for antioxidant defence pathways [79], although these readouts remain to be characterized in the blast fungus in the context of ferroptosis.

## Determining the relationship between conidial autophagy and ferroptosis in *M. oryzae*

Numerous independent reports have provided evidence that conidial cell death requires autophagy and that this is essential for appressorium function and plant infection by the blast fungus [4, 28, 33, 35, 36, 61]. It is clear, for example, that in the absence of autophagy, even though an appressorium can develop, it is unable to repolarise and develop a penetration peg to rupture the plant cuticle [4, 28]. How autophagy is precisely linked to ferroptosis, however, is not yet clear and further evidence will be necessary to establish how autophagy is induced and maintained, and how the switch to regulated conidial cell death occurs.

In mammals, cargo-dependent autophagy plays a role in mediating ferroptosis. Iron is sequestered in an inactive state in ferritin [80] and the cargo-dependent autophagic degradation of ferritin, mediated by the nuclear receptor coactivator 4 (NCOA4), then liberates iron and induces oxidative stress [81]. Knocking out core components of the autophagy machinery therefore inhibits ferroptosis [80]. It is also known that autophagic degradation of lipid droplets suppresses ferroptosis [82], suggesting that autophagy within conidial cells may be necessary prior to the switch to regulated cell death. A key area of future investigation in *M. oryzae* will therefore be to identify determinants of ferroptosis and determine how they are temporally and spatially regulated during the onset of appressorium maturation. Shen and colleagues [70] have demonstrated that both iron accumulation and lipid peroxidation occur within the distal conidium cell first prior to moving to adjacent cells, consistent with the order of cell death. Propagation of ferroptosis to neighbouring cells has been observed in other eukaryotic systems[72, 83, 84]. While the propagation of ferroptosis occurs independently of cell rupture, cell rupture hastens its spread [72]. Thus, a “pro-death” signal advances to neighbouring cells prior to cell death. Such a signal remains elusive to identify but may involve the spread of lipid peroxides or iron. How such a “pro-death” signal is transmitted between individual cells of the *M. oryzae* conidium is unclear, but septal pores do remain open during the onset of autophagy, prior to cell death. It is therefore possible that the temporal onset of autophagy is necessary to condition each conidial cell for ferroptosis, rather than a mobile signal. One possibility could be, for example, that autophagy provides the requisite iron concentration to enable ferroptosis to proceed. The source of iron liberated by autophagy to mediate conidium cell death, however, is also unknown. While the blast fungus lacks ferritin-like complexes, cargo-dependent autophagic liberation of intracellular iron is a pre-requisite for conidium cell death [70]. An alternative mechanism would involve metallothioneins, a class of iron-binding proteins, known to suppress lysosomal membrane permeabilization to protect against harm. It has been shown that autophagic flux directs cytoplasmic metallothioneins to the lysosome where they chelate redox-active iron in response to tumour necrosis factor and cycloheximide [85]. Vacuolar re-localization of metallothioneins may protect against ferroptosis by sequestering iron [85], which could explain why constitutive autophagy inhibits cell death. Interestingly, the Mmt1 metallothionein from *M. oryzae* is necessary for appressorium function and pathogenesis, but its role has not been fully characterized and it was previously proposed to play a role in appressorium cell wall differentiation [86]. Re-investigating *Δmmt1* mutants for a role in the regulation of ferroptosis may therefore prove to be revealing.

## Conclusions

Plant infection by the rice blast fungus requires the precise orchestration of cell division, autophagic recycling and regulated cell death to enable a functionally competent appressorium to breach the leaf surface. Precise cell-cycle checkpoints govern appressorium morphogenesis in response to the surface signals perceived by the fungus, including surface hardness, hydrophobicity and the absence of a rich source of exogenous nutrients, and it is this combination of factors that leads to induction of appressorium development, regulated most notably by the Pmk1 MAP kinase pathway. It is also clear that autophagy in the conidium is required for appressorium maturation and re-polarization and that this is necessary, but perhaps not sufficient, for cell death to occur. The recent reports of ferroptotic cell death suggest that it too is a pre-requisite for appressorium maturation and competence [70]. Understanding the interplay between autophagy and ferroptosis is therefore a critical line of future investigation. The precise role of the TORC1 and TORC2 complexes in regulating homoeostatic control of autophagy and cell growth is also likely to be pivotal to understanding plant infection and will require further analysis to build on previous studies [27]. A critical balance of autophagy is required, for example, to mediate appressorium maturation and cell death, which is consistent with a role for TOR signalling. Finally, it will be necessary to determine whether a threshold of cellular recycling, mediated by autophagy and trafficking to the incipient appressorium is an essential pre-requisite for regulated cell death to be activated. Given that both processes are absolutely essential for plant infection to occur, such an analysis will be vital and, importantly, may also provide new targets for disease intervention, which are urgently required.

## Supplementary information

Nature Publishing Group Checklist
